# Poor CD4^+^ T Cell Immunogenicity Limits Humoral Immunity to *P. falciparum* Transmission-Blocking Candidate Pfs25 in Humans

**DOI:** 10.3389/fimmu.2021.732667

**Published:** 2021-09-30

**Authors:** Marija Zaric, Arianna Marini, Carolyn M. Nielsen, Gaurav Gupta, David Mekhaiel, Thao P. Pham, Sean C. Elias, Iona J. Taylor, Hans de Graaf, Ruth O. Payne, Yuanyuan Li, Sarah E. Silk, Chris Williams, Adrian V. S. Hill, Carole A. Long, Kazutoyo Miura, Sumi Biswas

**Affiliations:** ^1^ Nuffield Department of Medicine, The Jenner Institute, University of Oxford, Oxford, United Kingdom; ^2^ Laboratory of Malaria and Vector Research, National Institute of Allergy and Infectious Disease, National Institutes of Health, Rockville, MD, United States; ^3^ NIHR Clinical Research Facility, University Hospital Southampton NHS Foundation Trust and Faculty of Medicine, University of Southampton, Southampton, United Kingdom

**Keywords:** CD4+, Pfs25, *P. falciparum*, transmission blocking, vaccine

## Abstract

*Plasmodium falciparum* transmission-blocking vaccines (TBVs) targeting the Pfs25 antigen have shown promise in mice but the same efficacy has never been achieved in humans. We have previously published pre-clinical data related to a TBV candidate Pfs25-IMX313 encoded in viral vectors which was very promising and hence progressed to human clinical trials. The results from the clinical trial of this vaccine were very modest. Here we unravel why, contrary to mice, this vaccine has failed to induce robust antibody (Ab) titres in humans to elicit transmission-blocking activity. We examined Pfs25-specific B cell and T follicular helper (Tfh) cell responses in mice and humans after vaccination with Pfs25-IMX313 encoded by replication-deficient chimpanzee adenovirus serotype 63 (ChAd63) and the attenuated orthopoxvirus modified vaccinia virus Ankara (MVA) delivered in the heterologous prime-boost regimen *via* intramuscular route. We found that after vaccination, the Pfs25-IMX313 was immunologically suboptimal in humans compared to mice in terms of serum Ab production and antigen-specific B, CD4^+^ and Tfh cell responses. We identified that the key determinant for the poor anti-Pfs25 Ab formation in humans was the lack of CD4^+^ T cell recognition of Pfs25-IMX313 derived peptide epitopes. This is supported by correlations established between the ratio of proliferated antigen-specific CD4^+^/Tfh-like T cells, CXCL13 sera levels, and the corresponding numbers of circulating Pfs25-specific memory B cells, that consequently reflected on antigen-specific IgG sera levels. These correlations can inform the design of next-generation Pfs25-based vaccines for robust and durable blocking of malaria transmission.

## Introduction

While several stages of the malaria parasite life cycle can be targeted with vaccination, a promising possibility for malaria elimination and eradication is the development of transmission-blocking vaccines (TBVs). TBVs work by inhibiting parasite development in the mosquito midgut through vaccine-elicited Abs taken up during the blood meal and, therefore, require a high level of antigen-specific Abs to be maintained over time (1–4). Among several TBV candidates, *Plasmodium falciparum* protein Pfs25 is the most clinically advanced. Pfs25 is expressed on the surface of zygotes during their development into ookinetes, and as this process takes place exclusively within the mosquito, this antigen is not expressed in the human host (3). Moreover, Pfs25 is highly conserved and a direct correlation between anti-Pfs25 IgG titers and transmission-blocking activity (TRA) has been established in animal models, making it an attractive antigen for a TBV development (1, 5).

In general, monomeric Pfs25 protein has been shown to be poorly immunogenic, but protein formulation and multimerization methods have been able to increase Ab titres (6–10). Here, we have applied the IMX313 technology, based on a chimeric version of the oligomerization domain from chicken complement inhibitor C4-binding protein (C4 bp), in order to obtain homogenous, self-assembling oligomers of Pfs25. This C4 bp oligomerization domain has been shown to spontaneously form soluble heptameric structures when expressed in *E. coli* (11) and we have already demonstrated in mice that Pfs25 fused to IMX313 domain improved Ab responses over the same amount of monomeric antigen when expressed in ChAd63 and MVA viral vectors (10). Although this approach showed great promise in mice (10), relatively low anti-Pfs25 Ab titers were generated in humans in our Phase I First-in-Human clinical trial (12). In this study, the transmission reducing activity of the antibodies generated was weak, but both test vaccines were well tolerated and demonstrated a favourable safety profile in malaria-naive adults.

Achieving high levels of Abs able to block parasite development within mosquito likely depends on the establishment and maintenance of antigen-specific B cells that upon antigen encounter proliferate and/or terminally differentiate into plasma cells or memory B cells, which seed the bone marrow and provide a lasting source of serum Abs. Critical to efficient stimulation and robust proliferation of antigen-specific B cells after vaccination and consequent affinity maturation of produced antigen-specific Abs is the initiation of germinal centre (GC) activity. Central to the GC reaction and somatic hyper-mutation (SHM) is the interaction of GC B cells with GC T follicular helper (Tfh) cells (13, 14). GC Tfh cells are both required and limiting for the GC reaction (15, 16). GC Tfh cells control the number of GC B cell divisions and therefore, the amount of SHM by individual GC B cell clones (17), impacting both quantity and quality of Ab response. GC Tfh cells express the chemokine receptor CXCR5, which guides their migration into B cell follicles in response to the CXCL13 ligand, as well as inducible costimulator (ICOS), which potently promotes class-switching and B cell differentiation into plasma cells and memory B cells (18–20). A subset of circulating CD4^+^CXCR5^+^ T cells which share both phenotypic and functional properties with GC Tfh cells has been identified in both humans and mice; ongoing GC reactions in peripheral lymph nodes result in the peripheral blood emergence of activated CD4^+^CXCR5^+^ Tfh cells, characterized by high expression of ICOS and programmed cell death protein 1 (PD1) in peripheral blood (20, 21).

In order to better understand the differences in humoral responses, we attempted to gain deeper mechanistic insights into the development of antigen-specific immune responses in both mice and humans after vaccination with ChAd63 and MVA, encoding Pfs25-IMX313. An N-terminal secretion signal peptide was fused to Pfs25-IMX313 to ensure secretion; vaccination was delivered intramuscularly, in a heterologous prime (ChAd63) – boost (MVA) regimen, with an 8-week interval. We focused on the development of antigen-specific B cell and Tfh cell responses and correlations of each with humoral immunogenicity. After exploring both B cell-intrinsic (specificity, frequency, phenotype, proliferative capacity), and extrinsic factors (CD4^+^ T cell help, Tfh cell availability and specificity) in mice and humans, we found that both correlate with vaccine-induced Ab titres and contribute to the superiority of anti-Pfs25 Ab responses in mice over humans.

## Results

### Murine Vaccine-Induced Abs Demonstrate Superior Transmission-Reducing Activity to Human

**Figure 1:**
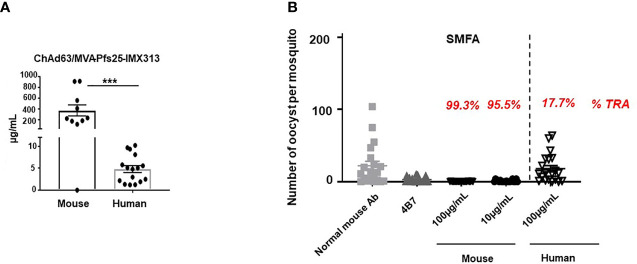
Murine vaccine-induced antibodies demonstrate superior transmission-reducing activity to human. **(A)** Anti-Pfs25 serum total IgG responses for murine and human samples, as assessed on d72 post ChAd63/MVA Pfs25-IMX313 vaccination. Statistical analysis using 2-tailed Mann-Whitney test ***p < 0.001. **(B)** Transmission-reducing efficacy of human and murine anti-Pfs25 IgG induced by the ChAd63/MVA Pfs25-IMX313. Total Pfs25-specific IgG was purified from either pooled murine or individual human serum obtained on d72 post vaccination. The purified Pfs25-specific human and murine IgG samples were mixed with *P. falciparum* NF54 cultured gametocytes, at 100µg/ml and fed to **(A)** stephensi mosquitoes (n = 20 per test group). Murine samples were also tested at a lower concentration of 10µg/ml. IgG from naive mice was used as a negative control (“normal mouse Ab”); the transmission blocking anti-Pfs25 mAb 4β7 was used as a positive control. Midguts were dissected 8 days post-feeding. Transmission reducing activity (%TRA) for human and murine test IgG samples was calculated relative to the negative control IgG tested in the same assay and is indicated in red above each corresponding sample. Vertical dashed line separates human from murine samples. Data are from one out of three independent experiments giving similar results.

BALB/c mice were primed (Day 0) with 1 × 10^8^ infectious units (vp) of ChAd63-Pfs25-IMX313 and boosted 8 weeks later (day 56) with 1 × 10^7^ plaque forming units (pfu) of MVA-Pfs25-IMX313, while the healthy UK adult volunteers enrolled into the VAC062 trial (ClinicalTrials.gov Identifier : NCT02532049) were primed with 5 × 10^10^ vp ChAd63 Pfs25-IMX313 followed by either 1x10^8^ pfu or 2x10^8^ pfu MVA Pfs25-IMX313 boost (Groups 2B and 2C, respectively) (12). Sera were collected from both mice and human volunteers on days 72 (2 weeks post-boost) and magnitude of the anti-Pfs25 serum IgG Ab response was assessed by ELISA against Pfs25 recombinant protein. Responses are reported in μg/ml following conversion of ELISA arbitrary units (AU) by calibration-free concentration analysis (CFCA), as direct comparison between murine and human anti-Pfs25 Ab responses detected by ELISA could not be performed, due to different sensitivities of the secondary Abs used. Vaccination using the same ChAd63/MVA Pfs25-IMX313 regimen produced significantly higher serum anti-Pfs25 IgG responses in mice compared to humans (**Figure 1A**). To test the possibility that this difference is due to the discrepancy in vaccine dose/body weight ratio between mice and humans, we i.m. immunized mice with full dose (1 × 10^8^ vp of ChAd63-Pfs25-IMX313 and 1 × 10^7^ pfu of MVA-Pfs25-IMX313), half-dose (5 × 10^7^ vp of ChAd63-Pfs25-IMX313 and 5 × 10^6^ pfu of MVA-Pfs25-IMX313) or tenth-dose (1 × 10^7^ vp of ChAd63-Pfs25-IMX313 and 1 × 10^6^ pfu of MVA-Pfs25-IMX313) of the same viral-vectored vaccine. Indeed, Ab responses to Pfs25 were substantially inferior to half-dose and tenth-dose as comparied to the full dose of the equivalent viral-vectored vaccine (**Supplementary Figures 1A, B**).

However, when the equal concentrations of murine and human Pfs25-specific IgG generated in response to viral vector vaccination was tested for functional activity in standard membrane feeding assay (SMFA) (22), murine antigen-specific Abs showed significantly higher transmission reducing activity (TRA) compared to human (**Figure 1B**, **Supplementary Table 1**). In this assay, total IgG was first purified from murine and human sera *via* Protein G affinity chromatography, followed by affinity purification of Pfs25-specific IgG using chromatography columns loaded with Pfs25 antigen. Equal concentrations of murine and human Pfs25-specific IgG were then tested in the SMFA and TRA was determined as the reduction in the number of oocysts compared to a negative control lacking protective Ab. When tested at a concentration of 100μg/ml, human anti-Pfs25 Abs did not demonstrate significant TRA, while, in the same assay, the equivalent concentration of antigen-specific Abs obtained from mice immunized with the identical vaccine displayed more than 99% TRA. Moreover, murine Pfs25-specific IgG showed statistically significant TRA when tested at the concentration as low as 10 μg/ml (**Figure 1B**), confirming that ChAd63/MVA Pfs25-IMX313 vaccination induces qualitatively superior Ab responses in mice compared to humans.

We also confirmed that the functional activity of murine anti-Pfs25 Abs tested in the SMFA was not influenced by the vaccine dose given. Purified total murine IgGs obtained from mice immunized with either full, half or tenth-dose of the same viral vectored vaccine all displayed more than 99% TRA when tested at concentrations of 350μg/ml and 125μg/ml in the SMFA (**Supplementary Figure 1C**).

### Differences in the Effector Functions of Induced Murine and Human Ab Subclasses, Ab Avidity and Cross-Reactivity Do Not Explain Qualitative Superiority of Murine Pfs25-Specific Abs

**Figure 2:**
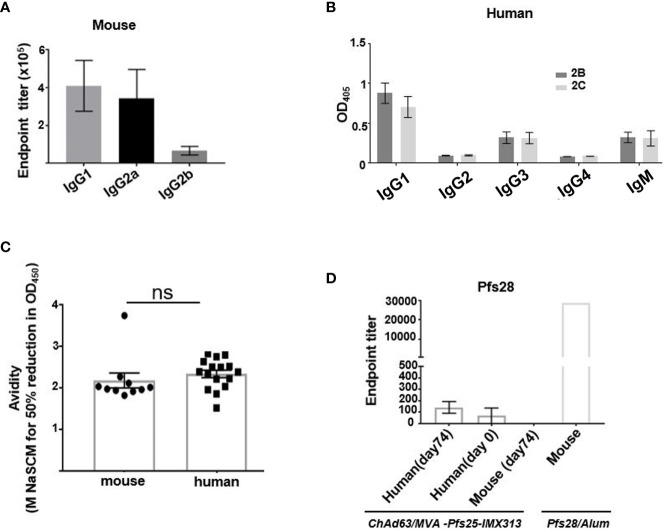
Assessment of murine and human antibodies’ effector functions, avidity and cross-reactivity. **(A)** Isotype profiles of murine Pfs25-specific antibody responses on d84 were assessed by ELISA. Responses are shown for IgG1, IgG2a and IgG2b. **(B)** Isotype profiles of human serum antibody responses were assessed by ELISA. Responses are shown for immunization groups 2B and 2C, assessed at d84 for IgG1, IgG2, IgG3, IgG4 and IgM. **(C)** Avidities of murine and human serum IgG responses at day 84 were assessed by NaSCN-displacement Pfs25 ELISA and are reported as the molar (M) concentration of NaSCN required to reduce the starting OD in the ELISA by 50% (IC50) Differences in avidity were assessed by Mann-Whitney test. Each symbol represents an individual response (ns- not significant). **(D)** Endpoint anti-Pfs28 IgG titers were assessed by ELISA on human serum samples isolated on d0 (n = 16) and d74 (n = 16) or murine samples (n = 6) obtained on d74 post ChAd63/MVA Pfs25-IMX313 vaccination. Murine serum sample acquired 2 weeks post boost immunization with recombinant Pfs28 protein in Alum, was used as positive control. Error bars show SEM.

To explore the reasons behind functional superiority of murine vaccine-induced Ab responses over Abs generated in humans, we investigated the IgG subclasses and avidity of Abs induced after viral-vector vaccinations in mice and humans. Out of all IgG subclasses tested, ChAd63/MVA Pfs25-IMX313 vaccination in mice induced mainly IgG1 and IgG2a responses as measured by ELISA at d84 (**Figure 2A**), while in humans the vaccine-induced serum Ab response against Pfs25, also assessed at d84, was composed of IgG1,IgG3 and IgM with little to no IgG2 or IgG4 (**Figure 2B**). Typically, human IgG1 and IgG3 are the most potent triggers of effector mechanisms, opposite to murine IgG1, which functionally resembles human IgG4 and does not interact with complement factors (23). Murine-induced antibodies against Pfs25 have been demonstrated to achieve transmission blocking in the SMFA in a complement-independent manner (8), but as IgG1 was the dominant subclass induced in human volunteers after ChAd63/MVA Pfs25-IMX313 vaccination, we still examined the possibility that complement could play an important role in improving the TRA of human anti-Pfs25 IgG in the SMFA. We found that addition of complement did not impact the TRA in the SMFA when it was added to three individual human samples with the highest anti-Pfs25 IgG1 titres (**Supplementary Figure 2**).

The avidity of the anti-Pfs25 IgG, as measured by a sodium thiocyanate (NaSCN) displacement ELISA, was comparable at d84 for both murine and human sera samples (**Figure 2C**).

Pfs28, another transmission-blocking target antigen, and Pfs25 are genetically linked and structurally similar (24). Moreover, these two proteins have been demonstrated to have multiple and partially redundant functions during ookinete/oocyst development in *Plasmodium berghei* parasites (25). We therefore tested the hypothesis that viral vector vaccination in mice, but not in humans, generated anti-Pfs25 Abs that were cross-reactive to Pfs28, contributing to the enhanced TRA in the SMFA of murine over human anti-Pfs25 IgG. Anti-Pfs25 Abs cross-reactive to Pfs28 were detected in neither murine nor human serum samples (**Figure 2D**). Overall, we concluded that qualitatively more efficient Ab response induced in mice compared to humans after ChAd63/MVA Pfs25-IMX313 vaccination could not be attributed to differences between murine and human antigen-specific Ab avidity, antigen cross-reactivity or differences in the effector functions of induced Ab subclasses.

### Superior Pfs25-specific B Cell Responses Are Induced in Mice Compared to Human Volunteers

**Figure 3:**
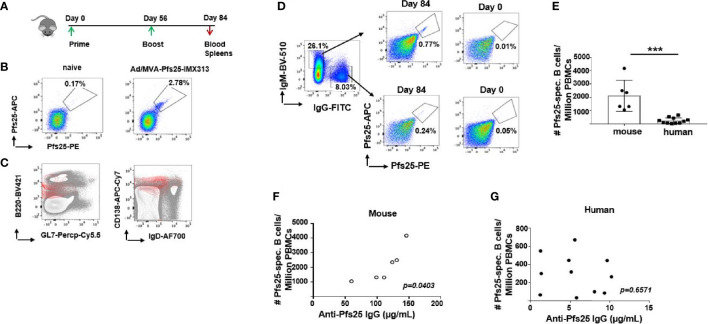
Differences in murine and human Pfs25-specific B cell responses. **(A)** Experimental design in mice. **(B)** Representative FACS plots showing frequencies of Pfs25-specific B cells accessed among PBMCs isolated from naïve or ChAd63/MVA Pfs25-IMX313 vaccinated mice on d84. **(C)** Flow cytometry contour plots of B220 *versus* GL7 (left panel) or CD138 *versus* IgD (right panel) expression on PBMC suspensions of mice immunized with ChAd63/MVA Pfs25-IMX313 (gray contours). Pfs25-specific B220^+^ cells are represented as an overlay (red contours) to signify that the majority of antigen-specific B cells were indeed B220^+^,GL7^-^,CD138^+^,IgD^low^. **(D)** Representative FACS plots showing frequencies of Pfs25-specific B cells among total IgM- or IgG-expressing memory B cells from PBMCs of trial vaccinees isolated on d0 and d84 post ChAd63/MVA Pfs25-IMX313 vaccination. **(E)** Bar graphs summarizing total numbers of Pfs25-specific B cells detected among individual murine and human PBMCs on d84 post vaccination. Data represent the mean ± SEM. Mann-Whitney test 2-tailed was performed to compare the two groups: ***p < 0.001. Correlations between serum anti-Pfs25 IgG concentrations and corresponding numbers of circulating antigen-specific B cells assessed for murine **(F)** and human **(G)** samples. P values were calculated using nonparametric Spearman’s test and are shown in the graphs.

For the direct assessment of Pfs25-specific immunity at the B cell level, we examined the frequency and specificity of B cells by flow cytometry using Pfs25 probes, within PBMC isolated from mouse and human blood on d84 post ChAd63/MVA Pfs25-IMX313 vaccination (**Figure 3A**, gating- **Supplementary Figures 3A, B**). We detected a sizable population of B cells that were Pfs25-specific among murine PBMCs (**Figure 3B**), with the majority comprising Ab-secreting cells, based on their B220^+^,GL7^-^, CD138^+^, IgD^low^ phenotype (**Figure 3C**). This population was also detectable within the spleens of immunized mice on d84 (**Supplementary Figure 3C**). Moreover, Pfs25 probes allowed us to confirm expansion of antigen-specific total and GC B cell responses (B220^+^, Fas^+^, GL7^+^) in murine popliteal (immunization-site draining) lymph nodes (LNs), as the proportion of antigen-specific B cells significantly increased at d14 compared to d7 (**Supplementary Figure 3D**).

Antigen-specific B cells were also identified in human PBMC samples (**Figure 3D**, gating- **Supplementary Figure 3B**), with considerably higher numbers of vaccine-induced antigen-specific IgM^+^ B cells compared to their IgG^+^ counterparts (**Supplementary Figure 3E**). Expectedly, the presence of Pfs25-specific B cells was observed neither in mouse nor in human d0 samples (**Figures 3B, D**). Among circulating PBMCs, we enumerated significantly larger population of the Pfs25-specific B cells in murine compared to human samples (**Figure 3E**). Furthermore, this population of Pfs25-specific B cells detected within the blood of immunized mice by flow cytometry correlated with serum levels of IgG Abs specific for Pfs25 measured on d72 post vaccination (r =0.8313; *p* = 0.0403) (**Figure 3F**). Such correlation was not evident for human samples (*r* =0.1512; *p* = 0.6571) (**Figure 3G**). Taken together, our data show that Pfs25 is markedly less immunogenic at both B cell and serological levels in humans compared to mice after ChAd63/MVA Pfs25-IMX313 vaccination.

### Superior Tfh Cell Activation Is Induced in Mice Compared to Human Subjects After ChAd63/MVA Pfs25-IMX313 Vaccination

Given that Tfh cells can be a controlling factor for B cell responses (26, 27), as they contribute to the development of Ab responses by providing help to memory B cells (21, 28), we wondered whether the poorer induction of Pfs25-specific B cells and Abs in humans following viral-vectored Pfs25-IMX313 is due to limiting Tfh responses. While direct measurement of GC responses in humans is often not feasible, a small population of circulating CXCR5^+^CD4^+^ T cells that express ICOS and high levels of PD1 share functional properties of GC Tfh cells and can be evaluated as proxies for GC Tfh cells (29).

**Figure 4:**
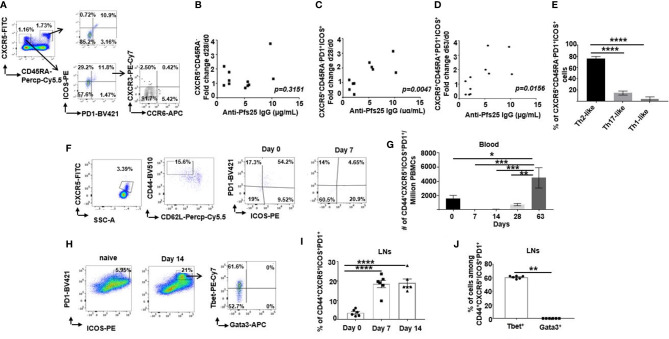
Differences in murine and human Tfh cell responses. **(A)** Flow cytometric plots of the frequency of ICOS^+^PD1^+^ cells among either CD4^+^CD3^+^CXCR5^+^CD45RA^+^ or CD4^+^CD3^+^CXCR5^+^CD45RA^-^ cells in the peripheral blood of healthy UK volunteers at d63 after ChAd63/MVA Pfs25-IMX313 vaccination. Flow cytometric assessment of the expression of CXCR3 and CCR6 within ICOS^+^PD1^+^CD4^+^CD3^+^CXCR5^+^CD45RA^-^ cells is also shown. Correlations are shown between serum anti-Pfs25 IgG concentrations measured on d72 post vaccination and corresponding changes in numbers of either circulating **(B)** CD4^+^CD3^+^CXCR5^+^CD45RA^-^ cells or **(C)** ICOS^+^PD1^+^CD4^+^CD3^+^CXCR5^+^CD45RA^-^ cells on d28 relative to d0, or corresponding changes in numbers of **(D)** ICOS^+^PD1^+^CD4^+^CD3^+^CXCR5^+^CD45RA^+^ cells on d63 relative to d0. P values were calculated using nonparametric Spearman’s test and are shown in graphs. **(E)** Bars summarising frequencies of CXCR3^-^CCR6^-^ (Th2-like), CXCR3^+^CCR6^-^(Th1-like) and CXCR3^- ^CCR6^+^ (Th17-like) Tfh cell populations, among ICOS^+^PD1^+^CD4^+^CD3^+^CXCR5^+^CD45RA^-^ cells as analyzed by flow cytometry. ****P < 0.0001, by one-way ANOVA, error bars show SEM. **(F)** Representative flow cytometric plots of the frequency of ICOS^+^PD1^+^ cells among CD4^+^CD3^+^CXCR5^+^CD44^+^CD62L^-^ cells in the peripheral blood of vaccinated mice assessed on d0 and d7 post vaccination. **(G)** Total numbers of ICOS^+^PD1^+^CD4^+^CD3^+^CXCR5^+^CD44^+^CD62L^-^ cells detected in peripheral blood of vaccinated mice at indicated time points. **(H)** Flow cytometry analyses of ICOS^+^PD1^+^CD4^+^CD3^+^CXCR5^+^CD44^+^CD62L^-^ cells isolated from popliteal LNs of either naïve (d0) or vaccinated mice at d7 post ChAd63 Pfs25-IMX313 immunization. Assessment of intracellular Tbet and Gata3 expression on those cells is also shown (right panel). Summarised data are shown in **(I)** and **(J)**. **P < 0.01, ****P < 0.0001, by Wilcoxon matched-pairs test, error bars show SEM.

Using flow cytometry, we assessed Tfh-like responses among cryopreserved PBMCs isolated from healthy individuals recruited to groups 2B and 2C of VAC062 trial on d0, d28 and d63 post ChAd63/MVA Pfs25-IMX313 vaccination (**Supplementary Figures 4A, B**). On d63, a population of circulating ICOS^+^PD1^+^ cells was detected among both memory (CD3^+^ CD45RA^-^CXCR5^+^CD4 ^+^) and naive/effector (CD3^+^CD45RA^+^CXCR5^+^CD4^+^) Tfh-like cellular compartments (**Figure 4A**, gating-**Supplementary Figure 4A**). Although a change in total memory CXCR5^+^CD4^+^ Tfh-like cell population detected on d28 relative to d0 did not correlate with the level of anti-Pfs25 IgG (*r =0.3373; p* = 0.3151) (**Figure 4B**), an increase in a subpopulation of those cells co-expressing ICOS and PD1 on d28 was associated with the sera concentration of antigen-specific IgG measured on d72 (*r* =0.7793; *p* = 0.0047) (**Figure 4C**), as well as with memory B cell responses measured by ELISPOT (r = 0.7427; p = 0.0088) (**Supplementary Figure 5A**). Similarly, the increase of effector ICOS^+^PD1^+^ among CD4^+^ CD45RA^+^ Tfh-like cells at d63, likely generated in a response to MVA Pfs25-IMX313 boost, correlated with both the quantity of specific Abs at d72 (*r* = 0.7039; *p* = 0.0156) (**Figure 4D**) and memory B cell responses measured by ELISPOT on d72 (r =0.6227; p =0.0407) (**Supplementary Figure 5B**). Furthermore, up-regulation of ICOS and PD1 expression was found to be mainly restricted to memory CXCR5^+^CD4^+^ T cells that did not express CXCR3 nor CCR6 (**Figures 4A, E**), indicating a commitment of vaccination-induced human blood Tfh cells to the Th2 pathway (21).

Extending these analyses to mice, we examined the frequency of ICOS^+^PD1^+^ among circulating Tfh cells (CD4^+^CXCR5^+^CD44^+^CD62L^-^) after ChAd63/MVA Pfs25-IMX313 vaccination (**Figure 4F**, gating-**Supplementary Figure 6A**). ICOS^+^PD1^+^ Tfh cells in blood were not detected at d7 and d14 post priming, but this population significantly increased between day 28 and d63 (**Figure 4G**), likely due to the fast recall response post MVA-Pfs25-IMX313 boost. Contrary to blood, this population was readily detectable in the popliteal LNs (**Figure 4H**, gating- **Supplementary Figure 6B**) on d7 after vaccination and was maintained until the d14 (**Figure 4I**), suggesting GC activation in the draining LNs after priming.

Several studies have shown that murine Tfh cells are capable of expressing Th1- or Th2-signature cytokines, which contribute to the regulation of B cell Ig isotype switching (21, 27, 30–32). Commitment of Tfh cells towards Th1-or Th2- signatures in mice is usually assessed by the expression of linage-specific transcription factors (33–35). Therefore, we examined the expression of T-bet and Gata3, the transcription factors crucial for differentiation of IFN-γ- and IL-4-producing Th1 and Th2 cells respectively **(** **Figure 4H**-right panel). ICOS^+^PD1^+^ Tfh cells isolated from the murine popliteal LNs on d14 post ChAd63/MVA Pfs25-IMX313 vaccination presented with the robust T-bet expression, with minimal-to-null Gata3 expression, committing them to Th1-like lineage (**Figure 4J**).Contrary to ICOS^+^PD1^+^ Tfh cells, Gata3 expression was readily detectable among total CD4^+^ T cells (**Supplementary Figure 6C**).

Overall, we showed that ChAd63/MVA Pfs25-IMX313 vaccination induced Tfh responses in both mice and humans; however, ICOS^+^PD1^+^ Tfh cells detected in healthy adult vaccinees demonstrated mainly Th2-like properties, while those in mice were of Th1-like cells, potentially explaining the differences in induced Pfs25-specific IgG subclasses and their corresponding Fc effector function profiles in mice and humans.

### Limited Antigen-Specific Tfh Response Is Generated in Humans Following Viral Vectored Pfs25-IMX313 Vaccination

**Figure 5:**
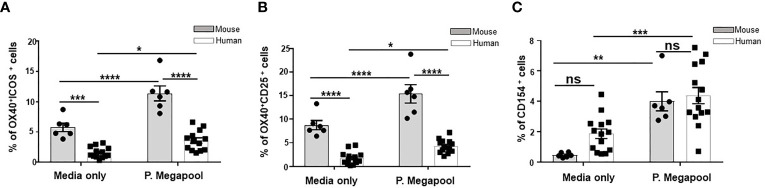
Assessment of antigen specificity of murine and human Tfh cells. Tfh cells were quantified among human PBMCs (n = 14) or murine splenocytes (n = 6) isolated on d14 after vaccination with ChAd63/MVA Pfs25-IMX313.Antigen-specific, among total Tfh cells were identified either by OX40 upregulation in combination with ICOS^+^ **(A)** or CD25 co-expression **(B)** or CD154 expression **(C)** following 18 hours of stimulation with media only or megapool of 20-mer synthetic peptide sets encompassing the Pfs-25-IMX313 insert (P. megapool). Error bars show SEM. ns - not significant, P > 0.05, *P < 0.05, **P < 0.01, ***P < 0.001, ****P < 0.0001, by Wilcoxon matched-pairs test.

To rule out the possibility that the Tfh cells we detected in both mice and humans were not generated mainly in a response to ChAd63 and MVA antigens, we stimulated cells from immunized Balbc mice and human vaccinees isolated on d63 post vaccination with overlapping 20-mer synthetic peptide sets encompassing the Pfs25-IMX313 insert (**Supplementary Figure 7**). Antigen-experienced human and murine Tfh were compared for the expression of the activation markers OX40, CD25, and/or ICOS, which preferentially identify antigen-specific Tfh cells compared to traditional intracellular cytokine staining method (36–38) (**Supplementary Figures 8A, B**, **Figures 5A, B**) or CD154 (CD40L) (**Supplementary Figure 8C**, **Figure 5C**), a classical marker of helper CD4^+^ T cells (39–41). *Ex vivo* assessment of both murine and human Tfh cell populations showed significantly greater expansion of peptide pool stimulated compared to unstimulated (media only) Tfh cells, irrespective of the surface marker combinations used to define antigen-specific Tfh cells (**Figures 5A-C**). Moreover, antigenic re-stimulation induced significantly larger proportion of murine Tfh cells co-expressing OX40, CD25, and/or ICOS compared to the corresponding human populations (**Figures 5A, B**). This was also evident when we similarly compared unstimulated (media only treated) human and murine Tfh cells in the same assay (**Figures 5A, B**), suggesting a possible higher proportion of endogenous vaccination-induced Tfh cells in mice compared to humans. Together, these results indicate that the induction of fewer human antigen-specific Tfh cells after ChAd63/MVA Pfs25-IMX313 vaccination may underpin the poorer immunogenic potential of Pfs25-IMX313 in humans compared to mice.

### Human Vaccine-Specific CD4^+^ T Cells Show Less Proliferative Capacity Compared to Murine Cells

**Figure 6:**
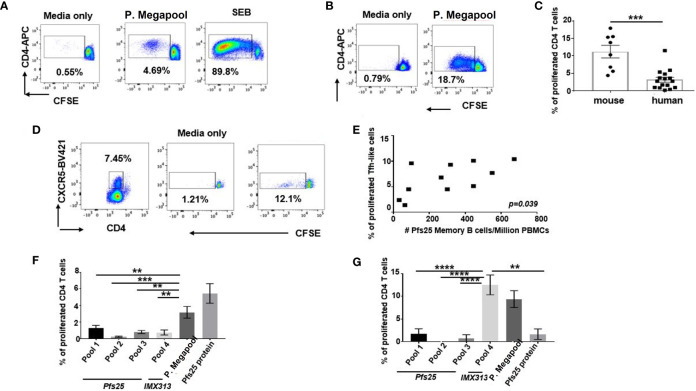
Proliferative capacity of vaccine-elicited murine and human CD4^+^ T cells. Cryopreserved and thawed human PBMCs **(A)** or murine splenocytes **(B)** isolated on d63 post vaccine administration were labelled with CFSE, stimulated with either media only or pool of stimulatory 20-mer peptides covering the entire Pfs25-IMX313 insert (P.megapool) for 6 days and analysed using flow cytometry for dividing (decreased CFSE) CD4^+^ T cells. As a positive control some cells were stimulated with staphylococcal enterotoxin B (SEB). Summarized data is shown in **(C)** Mann-Whitney test 2-tailed was performed to compare the two groups: ***p < 0.001. **(D)** Flow cytometric assessment of dividing human CXCR5^+^CD4^+^ cells in response to stimulation with media only or P. megapool is shown. **(E)** Correlation between total numbers of Pfs25-specific memory B cells (CD19^+^CD20^+^CD27^+^) detected on d84 among PBMCs of vaccinated healthy adult individuals and the percentage of their corresponding CD4^+^ T cells that divided CFSE in a response to P.megapool stimulation is shown. P value was calculated using nonparametric Spearman’s test and is shown in the graph. Percentage of human **(F)** and murine **(G)** CD4^+^ T cells that divided CFSE in a response to stimulation with either P. megapool, recombinant Pfs25 protein or separate stimulation with peptide pools 1-4,where pools 1-3 cover Pfs25 and pool 4 covers IMX313 portion of the vaccine construct. **P < 0.01, ***P < 0.001, ****P < 0.0001, by one-way ANOVA.

Exploring the potential reasons for the higher murine antigen-specific Tfh cellular responses over human elicited to the same vaccine, we assessed the differences in TCR specificities to Pfs25-IMX313 of human and murine CD4^+^ T cells, isolated on d63 post vaccination. Using CFSE proliferation assay, we analysed CFSE dilution on human or murine CD4^+^ T cells, 6 days after *ex vivo* recall stimulation with the pool of overlapping 20-mer synthetic peptide sets encompassing the Pfs-25-IMX313 protein (gating-**Supplementary Figures 9A, B**). The recall stimulation led to a proliferative response in both murine and human CD4^+^T cells (**Figures 6A, B**); however, significantly higher proliferative capacity to Pfs25-IMX313 peptides was detected with murine compared to human CD4^+^ T cells (*p* < 0.001) (**Figure 6C**). Among total human CD4^+^ T cells, we also assessed the proliferation of CXCR5-expressing Tfh-like cells (**Figure 6D**). Importantly, the rate of proliferated Tfh-like cells isolated from vaccinated volunteers on d63 was associated with the numbers of antigen-specific memory B cells, detected among their corresponding PBMCs on d84 post vaccination using Pfs25 probes (*r* = 0.6248; *p* = 0.039) (**Figure 6E**). In parallel, we also assessed the capacity of four separate peptide pools spanning the entire Pfs25-IMX313 vaccine insert (**Supplementary Figure 7**) to induce proliferation of murine and human CD4^+^ T cells. Lower frequencies of proliferated polyclonal human CD4^+^ T cells were detected in a response to separate stimulation with either of peptide pools 1-4, compared to frequencies induced by their cumulative stimulation, or stimulation with the entire Pfs25-IMX313 protein (**Figure 6F**, **Supplementary Figure 9C**). Surprisingly, in the same experimental settings, murine CD4^+^ T cells vastly and almost solely proliferated in response to pool 4, of which individual peptides cover only the IMX313 portion of the vaccine insert (**Figure 6G**, **Supplementary Figure 9D**).Together, these results suggest that the ChAd63/MVA Pfs25-IMX313 vaccination induces antigen-specific CD4^+^ T cell responses that differ in both strength and specificity between mouse and human. While, likely due to HLA polymorphism, no dominant epitopes derived from Pfs25-IMX313 vaccine could be identified for human CD4^+^ T cells, the exclusive specificity of murine CD4^+^ T cells were found to be to epitopes derived from IMX313, but not from the Pfs25 portion of the immunogen. This significantly weaker specificity to vaccine derived epitopes observed with human CD4^+^ T cells is likely a contributing factor for insufficient Pfs25-specific B cell responses observed after ChAd63/MVA Pfs25-IMX313 vaccination.

### Increased Sera CXCL13 Is Detectable in Mice, but Not in Healthy Adult Vaccinees Following ChAd63/MVA Pfs25-IMX313 Vaccination

**Figure 7:**
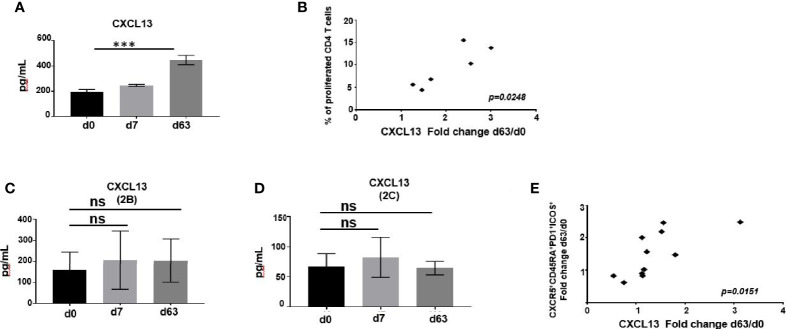
**(A)** CXCL13 was measured in murine serum following immunization at indicated time points by ELISA. Data represent mean ± SEM. Significance was calculated using ANOVA, ***P ≤ 0.001. **(B)** Correlation of change in CXCL13 levels on d63 relative to baseline with percentage of CD4^+^ T cells able to divide CFSE in a response to P.megapool stimulation. Correlation analysis performed using nonparametric Spearman’s test with 2-tailed P value, which is shown in the graph. CXCL13 was measured in serum of ChAd63/MVA Pfs25-IMX313 vaccinated trial individuals enrolled to groups 2B **(C)** or 2C **(D)** at indicated time points by ELISA. Data represent mean ± SEM. Significance was calculated using one-way ANOVA, ns-P > 0.05. **(E)** Correlation between changes in CXCL13 levels and numbers of ICOS^+^PD1^+^CD4^+^CD3^+^CXCR5^+^CD45RA^+^ cells both assessed on d63 post vaccination relative to baseline is shown. Correlation analysis performed using nonparametric Spearman’s test with 2-tailed P value, which is shown in the graph.

CXCL13, the chemokine ligand for CXCR5, is central in structuring GC development and has been described as a plasma biomarker for GC activity (42). We found that, relative to pre-vaccination status, the levels of CXCL13 in plasma of mice immunized with ChAd63/MVA Pfs25-IMX313 significantly increased when measured one week after the boost (**Figure 7A**, **Supplementary Figure 10A**) and that increase in CXCL13 levels for individual mice correlated with the corresponding degrees of CD4^+^ T cell proliferation, assessed by the CFSE proliferation assay (r =0.8684; p =0.0248) (**Figure 7B**).

Compared to pre-vaccination values, overall levels of CXCL13 detected in plasma of vaccinated volunteers enrolled to groups 2B and 2C were not significantly higher after priming with ChAd63-Pfs25-IMX313 or boosting with MVA-Pfs25-IMX313 (**Figures 7C, D**, **Supplementary Figure 10B**). When assessing individual CXCL13 responses among vaccinees, we observed that change in CXCL13 levels on d63 relative to d0 mirrored the change in total activated CD4^+^CXCR5^+^ICOS^+^PD1^+^ Tfh-like cell population detected on d63 after vaccination (r =0.8681; p =0.0151) (**Figure 7E**, **Supplementary Figure 10B**). Together, this greater induction of CXCL13 and antigen-specific Tfh cells in mice compared to humans imply increased GC activity in murine lymphoid tissues, promoting quantitatively and qualitatively superior antigen-specific Ab responses.

## Discussion

Various vaccine candidates targeting Pfs25 have been developed and shown to induce strong transmission-blocking activity in naive animal models (1, 7, 8, 10, 43–49). However, the identical Pfs25-based vaccines that were efficacious in mice (8, 50, 51), demonstrated limited success in clinical trials (52–54), highlighting the importance of better understanding the characteristics of humoral immunity to these Pfs25-based candidates in mice and humans to assess if it is the quantity or the quality of antibodies that is the limiting factor in achieving efficient transmission-blocking. Therefore, we performed a comparative study that, for the first time, characterized the immune responses to malaria TBV candidate ChAd63/MVA Pfs25-IMX313 in healthy adult volunteers and assessed differences in immunogenicity between mice and humans. We found that administration of Pfs25-IMX313 encoded by a replication-deficient ChAd63 and the attenuated MVA, elicited superior antigen-specific immune responses in mice over humans, generating higher Pfs25-specific CD4^+^ T cell and memory B cell responses and, ultimately, higher and more robust Ab titers and SMFA activity. The poor humoral immunogenicity of the Pfs25-IMX313 in humans was associated with an inability to efficiently elicit CD4^+^ T cell help after vaccination. This diminished vaccine-specific CD4^+^ T cell help in humans suggests that the Pfs25-IMX313 insert could be lacking in dominant MHC class II–restricted T cell epitopes as we observed that majority of human subjects weakly presented epitopes derived from Pfs25-IMX313 to CD4^+^ T cells. This can likely be attributed to heterozygosity of the human HLA class II type, as the outcome of many infectious diseases and vaccinations showed strong associations with particular HLA alleles (55, 56). Previously, when genetically outbred rhesus macaques were vaccinated with Pfs25 encapsulated into synthetic particles, mixed antigen-specific CD4^+^ T cell responses, ranging from modest to undetectable were reported (1). Intriguingly, in a response to viral-vector Pfs25-IMX313 vaccination, BalbC mice did not induce detectable Pfs25-specific CD4^+^ T cell responses, but strongly activated CD4^+^ T cells with the exclusive specificity to IMX313 domain of the vaccine. We have also previously demonstrated that Pfs25-IMX313 protein vaccine formulated in Alhydrogel or ASO1 readily induced a functional antibody response in outbred CD1 mice, although CD4^+^ T cell epitope specificity was not investigated in this study (47). The lack of T cell responses against Pfs25 was also observed in C57BL/6 mice, whose T cells were unable to bind Pfs25 *via* the I-Ab MHC class II molecule (45).

Although a poor Pfs25-specific Tfh cell response is probably not the exclusive reason explaining the weak Ab response to viral vectored Pfs25-IMX313 in outbred populations, the murine data suggest that the magnitude of the Tfh cell response could be a key factor in determining the immunogenicity of this vaccine. This information is important to consider while designing future Pfs25 based immunogens.

We do not exclude the possibility that observed differences in murine and human anti-Pfs25 responses may also be influenced by the differences in vaccine doses and subsequent limited availability of epitopes that could be seen by B cells, resulting in the weaker Ab responses in humans. Indeed, we observed a substantial reduction in the anti-Pfs25 IgG levels in mice immunized with half-dose of viral-vectored Pfs25-IMX313 vaccine. Nevertheless, even this lower quantities of antigen-specific IgG generated in a response to lower vaccine doses were still sufficient to demonstrate significant TRA in the SMFA. Moreover, when equal amounts of Pfs25-specific IgG isolated from murine and human sera were assessed for functional activity in the SMFA, murine antigen-specific Abs demonstrated superior function compared to human antibodies, likely suggesting the requirement for optimal Tfh responses to promote class switching and affinity maturation after vaccination consequently improving the quality of antigen-specific Abs.

Selective induction of Th1-like Tfh cells have been recognized as important for generating protective Ab responses in various human infection and immunization studies (57–60), while others recognized the beneficial role of non-Th1-like human Tfh cells in eliciting antigen-specific neutralizing Ab responses (61, 62). This polarization of Tfh cells depends on the stimuli induced by specific infections or immunizations, enabling Tfh cells to appropriately support B cell production of the Ab isotype required to clear the infection. In our study, Th1-like Tfh cell population was dominant after ChAd63/MVA Pfs25-IMX313 vaccination in mice, while the same vaccine induced Th2-biased Tfh responses in clinical trial vaccinees. This discrepancy could be reflected by the ability of differing environmental signals and innate immune cell responses to Pfs25-IMX313 in shaping Tfh cells differentiation in mouse and human lymphoid tissues. Indeed, significant differences between mice and humans in immune system activation and response to the same antigenic challenge, in both the innate and adaptive arms, have been identified, all likely impacted by the evolutionary distance between the species (63). For example, it has been recognized that in mice, IL-6 and IL-21 support Tfh cell differentiation, whereas IL-2 suppresses Tfh cells; differently, IL-12, with IL-23 and transforming growth factor-beta (TGF-β) support Tfh cells in humans (64–66). This opposing Tfh cell polarisation between mice and humans after ChAd63/MVA Pfs25-IMX313 vaccination was also mirrored in production of specific Ab isotypes with differing abilities to bind FcR or fix complement, suggesting that production of Th1-supported isotypes and the selective induction of Th1-like Tfh cells may be important for generating protective anti-Pfs25 transmission-blocking responses. As such, skewing Tfh cells away from Tfh2-like and toward Tfh1-like in humans may represent a potential avenue to enhance transmission reducing activity of Pfs25 Abs.

Using NaSCN displacement assay, we did not observe significant differences in avidities between murine and human anti-Pfs25 Abs. However, this assay measures binding strength of polyclonal Abs, but the determined avidity index does not always reflect affinity (67), and possible more finely dissected differences in monoclonal levels of murine and human Ab responses could not be assessed. Therefore, the alternative techniques, such as surface plasmon resonance and biolayer interferometry, are warranted to measure Ab-binding properties and evaluate true polyclonal vaccine-induced anti-Pfs25 Ab responses in mice and humans.

Pfs25-specific memory B cells were evident within our murine and adult human cohorts. Following immunization with viral vectored Pfs25-IMX313 vaccines, we observed an expansion of Pfs25-specific immunity at B cell levels, albeit to a lesser magnitude in human vaccinees compared to vaccinated mice, broadly consistent with magnitudes of both T helper cell and serum Ab responses. We found augmented GC activity over time in lymphoid tissues of animals vaccinated with Pfs25-IMX313 *via* viral vectors, as assessed by CXCL13, induction of Tfh cells, and detection of GC B cells by flow cytometry. In the clinical study, we did not have access to the vaccine draining LNs to evaluate GC formations and instead, used other measures for induction of GC activity (42). Contrary to mice, levels of CXC13 did not significantly increase in humans by priming with ChAd63 Pfs25-IMX313 or boosting with MVA Pfs25-IMX313, implying overall weak GC activity in human subjects. This was also supported by the finding that larger portion of antigen-specific B cells, detected among human PBMCs, expressed IgM compared to IgG. As during class-switching, occurring as a consequence of GC activation, B cells switch from surface IgM expression to IgG, IgA and IgE isotypes (68), our data suggest likely absence of adequate GC formations in humans after ChAd63/MVA Pfs25-IMX313 vaccination. While Pfs25-specific memory B cells are clearly targetable by vaccination in humans, the longevity of such responses remains unclear and further studies into strategies to extend the durability of Pfs25-specific immunity are warranted.

In summary, we found that the immunological dynamics of humoral immunity targeting the Pfs25-IMX313 domain are complex and extrapolating data from mice, we identified several ways immunogenicity to Pfs25 could be increased for clinical implementation. Our results demonstrate limited immunogenicity of the Pfs25 domain in humans after ChAd63/MVA Pfs25-IMX313 vaccination, a finding consistent with the already reported poor immunogenicity of this protein (52–54). We cannot rule out the idea that different Pfs25-directed vaccine approaches may have differing immunogenic potential, given the diverse range of immunogen designs, expression systems, formulations and immunization schedules. Although it is encouraging that vaccination of humans can induce transmission-blocking Abs targeting Pfs25 when delivered *via* viral vectors (12), it does seem likely, however, that boosting the immunogenicity of the Pfs25 *via* novel adjuvants, carrier proteins and nanoparticle or viral-particle formulations will be necessary to elicit robust titers of Pfs25-immunity in humans. In particular, it would be pertinent to consider formulations that contain CD4^+^ T cell epitopes and adjuvants that trigger pathways known to enhance Tfh cell differentiation, enabling them to guide an appropriate B cell response and achieve sufficient amount of high-quality Abs able to facilitate transmission blocking. Indeed, our results demonstrate that improving the quantity of antibodies to the Pfs25 antigen should not be only consideration when designing Pfs25-based TBVs. In fact, the quality of antibodies induced by vaccination is of key importance for the efficient malaria transmission blocking. Moreover, this is likely applicable to other vaccine candidates aiming to confer antibody-mediated protection against relevant pathogens.

## Material and Methods

### ChAd63 and MVA Pfs25-IMX313 Vaccines

The design, GMP production and preclinical testing of the viral vector vaccines used in this study have been reported previously (10). For the Pfs25-IMX313 constructs a 229 bp DNA fragment encoding the IMX313 domain was cloned at the C-terminus of Pfs25. The final antigen insert was codon optimized for mammalian expression and fused in frame to the human tissue plasminogen activator (tPA) leader sequence.

The Pfs25-IMX313 insert was subcloned into the ChAd63 and MVA destination and shuttle vectors. ChAd63 Pfs25-IMX313 was manufactured under current Good Manufacturing Practice (cGMP) conditions by the Clinical Biomanufacturing Facility (CBF), University of Oxford, UK, and MVA Pfs25-IMX313 was manufactured under cGMP conditions by IDT Biologika GmbH, Germany, both as previously described (69, 70).

### Proteins

Recombinant Pfs25 protein for ELISA and B cell assays was produced from a stably transfected Drosophila melanogaster Schneider 2 (S2) cell line. Pfs25 sequence (GenBank accession no: AAN35500, from Alanine-22 to Threonine-193) with three potential N-linked glycosylation sites (112, 165, 187) mutated, was codon optimized for expression in insect cells (GeneArt^®^ Life Technologies, Germany). Cell line generation and growth conditions have been previously described in detail (71, 72). Clarified supernatant from a 4-day batch culture of S2 cells was concentrated 15-20 fold and buffer exchanged using a TFF system fitted with Pellicon 3 Ultracel 3 kDa membrane (Merck Millipore, UK). Purification was performed on an AKTA Pure 25 system (GE Healthcare, UK), consisting of an affinity step with CaptureSelect™ C-tag column (Thermo Fisher Scientific, UK) and a polishing size exclusion chromatography (SEC) using Superdex 200 16/600 PG (GE Healthcare, UK) in 20 mM TrisHCl, 150 mM NaCl, pH 7.4 (TBS). Purified protein was quantified by Nanodrop (Thermo Fisher Scientific, UK) and stored at -80°C until further use.

IMX313 protein used for ELISA was kindly provided by OSIVAX who owns rights to the IMX313 vaccine technology.

### Participants

Healthy, malaria-naive males and non-pregnant females aged 18-50 years were invited to participate in the study. Volunteers were recruited and vaccinated at the CCVTM, University of Oxford and at the NIHR CRF in Southampton. In total, twenty six volunteers were enrolled, with twenty four vaccinated as per protocol and twenty two completing follow-up. This was a Phase I open-label, dose escalation, first-in-human, non-randomized trial of the viral vectored vaccines ChAd63 Pfs25-IMX313 and MVA Pfs25-IMX313 given in a prime-boost regimen with an eight week interval (12). The study received ethical approval from the Oxfordshire Research Ethics Committee A in the UK (REC reference 15/SC/0237). The study was also reviewed and approved by the UK Medicines and Healthcare products Regulatory Agency (MHRA, reference 21584/0344/001-0001). Volunteers signed written consent forms and consent was verified before each vaccination. The trial was registered on Clinicaltrials.gov (NCT02532049) and was conducted according to the principles of the current revision of the Declaration of Helsinki 2008 and in full conformity with the ICH guidelines for Good Clinical Practice (GCP).

### Animal Vaccination

All animal experiments and procedures were performed according to the UK Animals (Scientific Procedures) Act Project Licence (PPL PA7D20B85) and approved by the Oxford University Local Ethical Review Body. Age-matched female BALB/c mice, housed in specific-pathogen free environments, were vaccinated with equal amount of vaccines into each legs *via* the intramuscular route (i.m.) using a heterologous prime-boost viral-vectored regime, when mice were primed with 1 × 10^8^ i.u. ChAd63 and boosted 8 weeks later with 1 × 10^7^ pfu MVA. Vaccines were prepared in sterile endotoxin-free PBS (Sigma-Aldrich, UK).

### Peptides Used for Assessment of Ag-specific CD4^+^ T Cell Responses

20-mer peptides, overlapping by 10 amino acids, spanning the Pfs25 and IMX313 inserts (**Supplementary Figure 7**) were purchased from NEO Scientific (Cambridge, MA, USA) and used for assays as described.

### Total IgG ELISAs

ELISAs against Pfs25 recombinant protein were performed using standardized methodology as previously described for murine (10, 73) and human (69, 74) samples, except that plates were blocked with StartingBlock™ T20 (PBS) Blocking Buffer (ThermoFisher Scientific,UK).

For Pfs28 endpoint ELISA, Nunc-Immuno maxisorp plates were coated with recombinant Pfs28 protein, produced from the Drosophila S2 cells. Indicated serum samples were added in duplicates and diluted 3 fold down the plate, followed by the same procedure as for the Pfs25 standardized ELISA. Optical density (OD) was read at 405 nm using an ELx800 absorbance microplate reader (Biotek, UK). The endpoint titer is defined as the X-axis intercept of the dilution curve at an absorbance value ( ± three standard deviations) greater than the OD for a negative control serum sample.

### Avidity ELISAs

Ab avidity was assessed using a sodium thiocyanate (NaSCN)-displacement ELISA. Nunc-Immuno maxisorp plates were coated with recombinant Pfs25 protein over-night blocked and then washed with PBS/T as before. All individual serum samples were diluted so that each sample contained the same level of Pfs25 AU (in this study 1 AU). Samples were added in duplicate, following incubation and washing, an ascending concentration of the NaSCN (0–7M) was added to the wells. The plates were incubated at RT for 15 min followed by washing and further development as before. The avidity ELISA readout was the intercept of the curve (molar concentration of NaSCN/OD) where OD reached a 50% reduction of the OD in NaSCN-free samples.

### IgG Subclass ELISAs

For the IgG subclass endpoint ELISA, biotinylated anti-mouse IgG1 or IgG2a (BD bioscience) was used as the secondary Abs. After incubation and wash, alkaline phosphatase conjugated ExtrAvidin (Sigma-Aldrich, UK) was added at 1:5000 in PBS and incubated for 30 min at RT. The development and measurement of OD and the endpoint titer determination were done as before (10).

Human Ab isotype ELISAs were also performed using methodology described in detail elsewhere (70), except that plates were coated with recombinant Pfs25 produced from the Drosophila S2 cells at 2 μg/ml.

### CXCL13 ELISAs

Mouse and human CXCL13 serum concentrations were determined using LEGEND MAX™ Mouse CXCL13 (BLC) ELISA kit (BioLegend) and human CXCL13 (BLC) ELISA Kit (Life Technologies Ltd), respectively, as per manufacturers’ instructions.

### Calibration-Free Concentration Analysis (CFCA)

CFCA was performed as previously described (12, 70, 75), using a Biacore X100 instrument, a Biotin CAP chip, and X100 control and evaluation software (GE Lifesciences, UK). Briefly, CFCA was performed using serum samples isolated either from vaccinated mice or human subjects in Groups 2B and 2C with a range of Pfs25-specific IgG Ab responses as assessed by ELISA. Each volunteer’s serum was diluted 1:20, while individual murine serum samples were diluted 1:500 in running buffer (75). Mass-transport limited binding conditions were obtained by capturing a minimum of 3000 response units (RU) of Pfs25 antigen on the active flow cell. The CFCA-measured Pfs25-specific Ab concentrations for each individual volunteer were analysed by linear regression with the corresponding total IgG ELISA AU data, with the slope of the line used to derive an AU-to-µg/mL conversion. The same principle was applied to determine Pfs25-specific Ab concentrations in murine serum samples.

### Flow Cytometric Detection of Pfs25-Specific B Cells

Pfs25-specific B cells were identified within cryopreserved human PBMCs by costaining with Pfs25 probes. Probes were prepared freshly for each experiment by incubation of monobiotinylated Pfs25 with streptavidin-PE or streptavidin-APC at an approximately 4:1 molar ratio to facilitate tetramer generation (76, 77). The monoclonal Abs used for surface staining included the following: CD19-PE-Cy7 (HIB19), CD20-BV421(2H7), IgM-BV510 (MHM-88), CD27-Percp-Cy5.5 (O323) and IgG-FITC (HP6017), all from BioLegend). For murine splenocyte and LN samples, tissues were mechanically homogenized into single-cell suspensions in R10 media (RPMI 1640, 10% FCS, 1× penicillin-streptomycin-glutamine; Life Technologies, Thermo Fisher Scientific) and red blood cells lysed with ACK buffer. Murine cells (PBMCs, splenocytes and LN cells) were surface stained with the same Pfs25 probes and the following surface Abs: B220-BV421 (RA3-6B2); IgD-Alexa Flour700 (11-26c.2a); GL7-Percp-Cy5.5 (GL7); CD138-APC-Cy7 (281-2) and Fas-FITC (SA367H8), all from Biolegend. Cells were washed twice and acquired on a BD LSRII and data were analysed in FlowJo (version 10, Treestar).

### Flow Cytometric Detection of Activated and Antigen-Specific Tfh Cells

For *ex vivo* activated Tfh cell quantification, freshly isolated murine PBMC or spleen suspensions were stained with the following Abs: CD3-Alexa Fluor 700 (17A2); CD4^+^-APC-Cy7 (GK1.5); CXCR5-FITC (L138D7); CD4^+^4-BV510 (IM7); CD62L-Percp-Cy5.5 (MEL-14); ICOS-PE (C398.4A); PD1-BV421(29F.1A12); Tbet-PE-Cy7 (4B10); Gata3-APC (16E10A23), all from BioLegend, while corresponding human PBMC samples were stained with CD4^+^-APC-Cy7 (A161A1); CD3-Alexa Fluor 700 (HIT3a); CD4^+^5RA-Percp-Cy5.5 (HI100); CXCR5-FITC (J252D4); ICOS-PE (C398.4A); PD1-Pacific Blue (EH12.2H7); CCR6-APC (G034E3) and CXCR3-PE-Cy7 (G025H7), all from Biolegend.

To identify antigen-specific Tfh cells, human or murine cell samples were cultured in R10 media for 18 hours at 37°C. The samples were stimulated with a peptide pool (1 μg/peptide/ml) comprising the Pfs25 (17 peptides) and IMX313 domain (5 peptides) or with a Media/DMSO control. Peptide pools were generated from a Pfs25-IMX313 peptide array (20-mers overlapping by 10 amino acids). At the time of stimulation, an anti-mouse or anti-human–CD154-PE (MR1 and 2431; respectively, both from BioLegend) were added to all culture conditions. After stimulation, cells were washed twice in PBS and human cells stained with CD3-Alexa Fluor 700 (HIT3a); CD4^+^-BV421 (GK1.5); CXCR5-FITC (J252D4); OX40-APC (ACT35); ICOS-APC-Cy7 (C398.4A); CD25-PE-Cy7 (BC96); and mouse samples with CD3-Alexa Fluor 700 (17A2); CD4^+^-BV421 (GK1.5); CXCR5-Percp.Cy5.5 (L138D7); OX40-APC (OX86); ICOS-APC-Cy7 (C398.4A); CD25-PE-Cy7 (3C7), all from BioLegend before being washed and acquired on a BD LSR II using BD FACSDiva.

### CSFE Proliferation Assay

Cryopreserved human PBMCs or isolated mouse splenocytes were resuspended in pre-warmed PBS with 0.1% BSA at a final concentration of 10^6^ cells/ml and labelled with 750 nM CFSE (5(6)-Carboxyfluorescein diacetate N-succinimidyl ester; Molecular Probes™) for 10 min at 37°C, 5% CO_2_. The staining was quenched by adding 5 volumes of ice-cold R10 followed by a 5-min incubation on ice. The cells were pelleted, washed and plated in 96-well round-bottom plates at a concentration of 1x10^6^ cells/well. The CFSE-labelled cells were then stimulated with 1 μg/peptide/ml of indicated peptide pools, or with 5 μg/ml whole Pfs25 protein, or 1 μg/ml staphylococcal enterotoxin B (SEB) (positive control) and R10 (negative control) for 6 days, stained with a dead cell marker (LIVE/DEAD Fixable Aqua stain; Invitrogen) and anti-CD4^+^-APC (BioLegend), anti-CD3-Alexa-Fluor 700 (BioLegend) and anti-CD8-Alexa Flour 780 (Biolegend) mAbs and acquired on a BD LSR II flow cytometer. Data analysis was performed using FlowJo software (Tree Star Inc.)

### Antigen-Specific Ab Affinity Purification

Pfs25-specific human and mouse IgG samples were generated in two stages. Serum from immunized mice or healthy adult volunteers was diluted 2:1 in binding buffer and purification was performed on an AKTA Pure 25 system (GE Healthcare, UK) using HiTrap™ Protein G HP column (GE Healthcare, UK). Pfs25-specific IgG was then purified from the total IgG on an AKTA Pure 25 system (GE Healthcare, UK) using Pfs25-conjugated HiTrap NHS-Activated HP affinity columns (GE Healthcare, UK). These were generated using Pfs25 protein and according to the manufacturer’s instructions. After purification, three rounds of buffer exchange into PBS using an Amicon 10K MWCO centrifugal filtration device were performed.

### SMFA

The ability of vaccine-induced Abs to block the development of *P. falciparum* strain NF54 was evaluated using the SMFA as previously described (5). The percentage of mature Stage V gametocytes was adjusted to 0.15% ± 0.05% and the male-female ratio is stable (almost always 1 male: 2–3 female). The positive control mouse monoclonal Ab 4B7 was used at a concentration of 0.094 mg/mL. Gametocyte cultures mixed with samples were then fed to 4–6 day old starved female *Anopheles stephensi via* a parafilm^®^ membrane. The mosquitoes were maintained at 26°C and 80% relative humidity. After 8 days, midguts from twenty mosquitoes per group were dissected, oocysts counted and the number of infected mosquitoes recorded. Percent reduction in infection intensity was calculated relative to the respective control IgG tested in the same assay.

### Statistical Analysis

Data were analyzed using GraphPad Prism version 6.07 for Windows (GraphPad Software Inc., California, USA) and R (version 3.5.1). All tests used to determine significance are described in the figure legends. A value of *P* < 0.05 was considered significant.

## Data Availability Statement

Further information and request for data, resources and reagents should be directed to and will be fulfilled by the Lead Contact, Prof. SB (sumi.biswas@ndm.ox.ac.uk).

## Ethics Statement

The studies involving human participants were reviewed and approved by Oxfordshire Research Ethics Committee A in the UK (REC reference 15/SC/0237). The patients/participants provided their written informed consent to participate in this study. The animal study was reviewed and approved by the University of Oxford Local Ethical Review Body.

## Author Contributions 

Conceived and performed the experiments: MZ, AM, GG, KM, TP, SE, IT, HG, and RP. Intellectually contributed to the project: MZ, AM, CN, CL, AH, and SB. Analyzed the data: MZ, KM, and SB. Contributed reagents/materials/analysis tools: YL, DM, CN and SS. Project Management: IT. Wrote the paper: MZ, CW, and SB. All authors contributed to the article and approved the submitted version.

## Funding

This work was supported by the European Union Seventh Framework Programme (FP7/2007-2013) under the grant agreement for MultiMalVax (number 305282). The study was also supported in part by UK NIHR infrastructure through the NIHR Oxford Biomedical Research Centre (the views expressed are those of the authors and not necessarily those of the NHS, the NIHR or the Department of Health). The SMFA work was supported by the Intramural Research Program of NIAID, NIH and by PATH’s Malaria Vaccine Initiative. SJD, AVSH and SB are Jenner Investigators, and CMN is a Wellcome Trust Sir Henry Wellcome Postdoctoral Fellow (209200/Z/17/Z).

## Conflict of Interest

AH is named inventor on patent applications covering malaria vaccines and immunization regimens.

The remaining authors declare that the research was conducted in the absence of any commercial or financial relationships that could be construed as a potential conflict of interest. 

## Publisher’s Note

All claims expressed in this article are solely those of the authors and do not necessarily represent those of their affiliated organizations, or those of the publisher, the editors and the reviewers. Any product that may be evaluated in this article, or claim that may be made by its manufacturer, is not guaranteed or endorsed by the publisher.
