# Supplementary material for: Poor CD4+ T Cell Immunogenicity Limits Humoral Immunity to P. falciparum Transmission-Blocking Candidate Pfs25 in Humans
Source: Front Immunol. 2021 Sep 30;12:732667. doi: 10.3389/fimmu.2021.732667 (PMC8515144; doi:10.3389/fimmu.2021.732667)
Supplement: Supplementary file 1 [file Presentation_1.pdf]

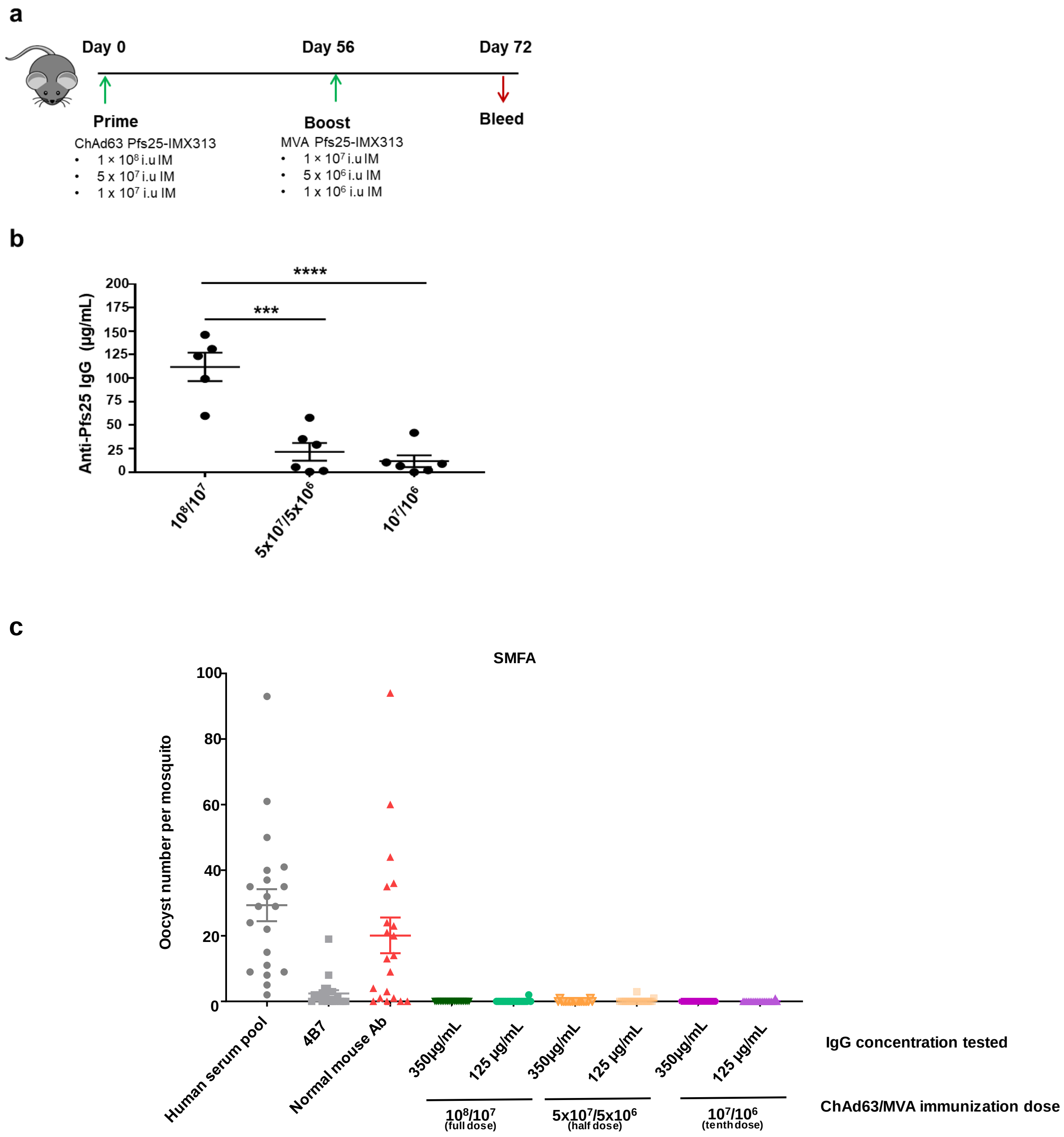

Supplementary Figure 1: Reducing the vaccine dose significantly diminishes antigen-specific antibody concentration but not quality. (a) *Experimental design in mice.* ***(b)*** *Anti-Pfs25 serum total IgG responses detected in mice immunized with full dose, half-dose or tenth-dose of the ChAd63/MVA Pfs25-IMX313 vaccine, as assessed on d72 post vaccination. Each symbol represents individual mouse. Statistical analysis using one-way ANOVA, ***P < 0.001, ****P < 0.0001.* **(c)** Total IgG was purified from *mice immunized with full dose, half-dose or tenth-dose of the ChAd63/MVA Pfs25-IMX313 vaccine* on d72 post vaccination. Murine IgG samples were mixed with *P. falciparum* NF54 cultured gametocytes, at 350µg/ml and 125µg/ml and fed to A. stephensi mosquitoes (n=20 per test group). Midguts were dissected 7 days post-feeding and oocyst number per mosquito enumerated.

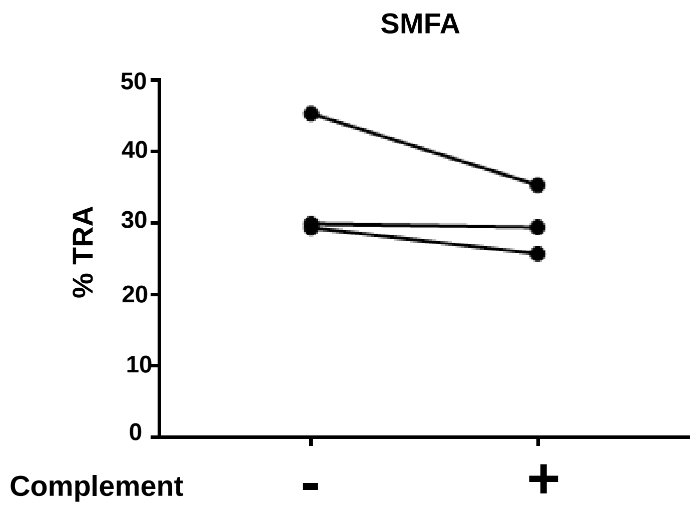

Supplementary figure 2: Addition of complement does not improve transmission-reducing activity of human antibodies in the SMFA. *Pfs25-specific IgG was purified from three individual human serum obtained on d72 post vaccination. The purified Pfs25-specific human IgG samples were mixed, with and without addition of complement, with P. falciparum NF54 cultured gametocytes, at 100µg/ml and fed to A. stephensi mosquitoes (n=20 per test group). Midguts were dissected 7 days post-feeding. Percent reduction in infection intensity was calculated relative to the negative control IgG tested in the same assay.*

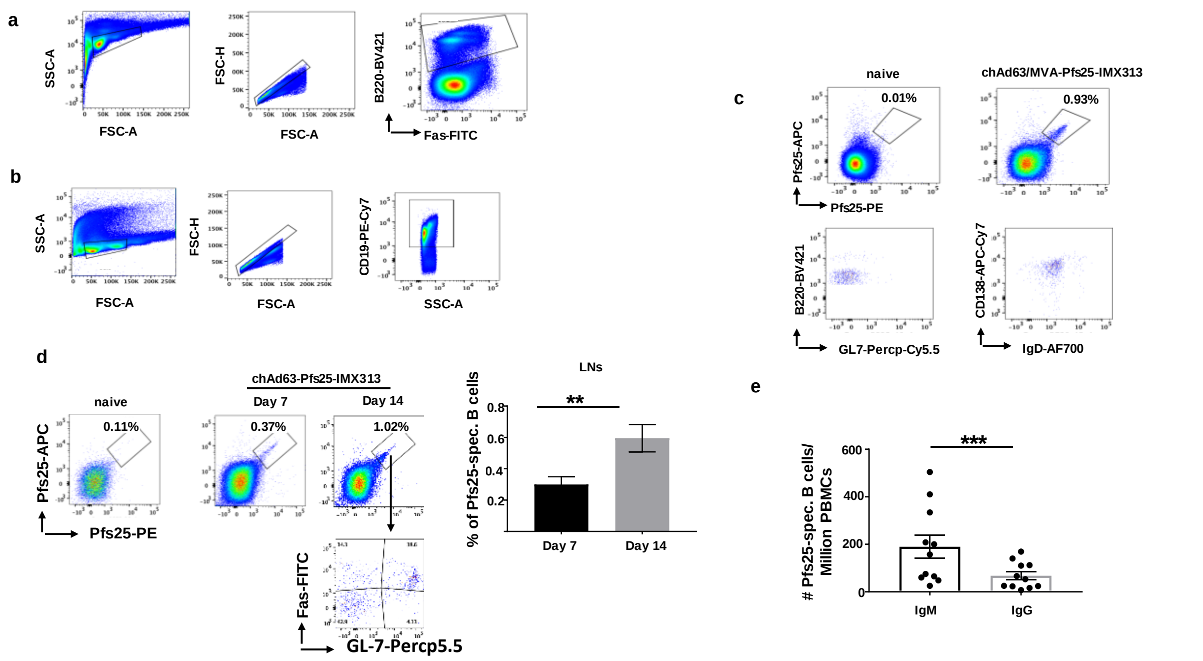

Supplementary Figure 3: Differences in murine and human Pfs25-specific B cell responses. (a) *Gating strategy used to identify B cells in murine blood.* ***(b)*** *Gating strategy used to identify B cells among human PBMCs.* ***(c)*** *Representative FACS plots showing frequencies of Pfs25-specific B cells accessed among spleen cell suspensions isolated from naïve or ChAd63/MVA Pfs25-IMX313 vaccinated mice on d84 (top panel). Flow cytometry plots of B220 versus GL7 (left) or CD138 versus IgD (right) expression on Pfs25-specific B cells isolated from spleen cell suspensions of mice immunized with ChAd63/MVA Pfs25-IMX-313.* ***(d)*** *Representative FACS plots showing frequencies of Pfs25-specific B cells accessed among popliteal LN cell suspensions isolated from naïve or ChAd63 Pfs25-IMX313 vaccinated mice on d7 and d14 post immunization. Flow cytometry contour plots showing Fas versus GL7 expression on Pfs25-specfic B cells isolated from murine popliteal LN cell suspensions on d14 post of ChAd63/MVA Pfs25-IMX313 vaccination. Bar graphs show enumerated data. Statistical significance between groups was assessed by Mann-Whitney test, **P < 0.01, ns=P>0.05.* ***(e)*** *Numbers of Pfs25-specific memory B cells that were either IgM^{+} or IgG^{+}, detected among human PBMCs on d84 post ChAd63/MVA Pfs25-IMX313 vaccination. Statistical significance between groups was assessed by Man-Whitney test, ***P < 0.001.*

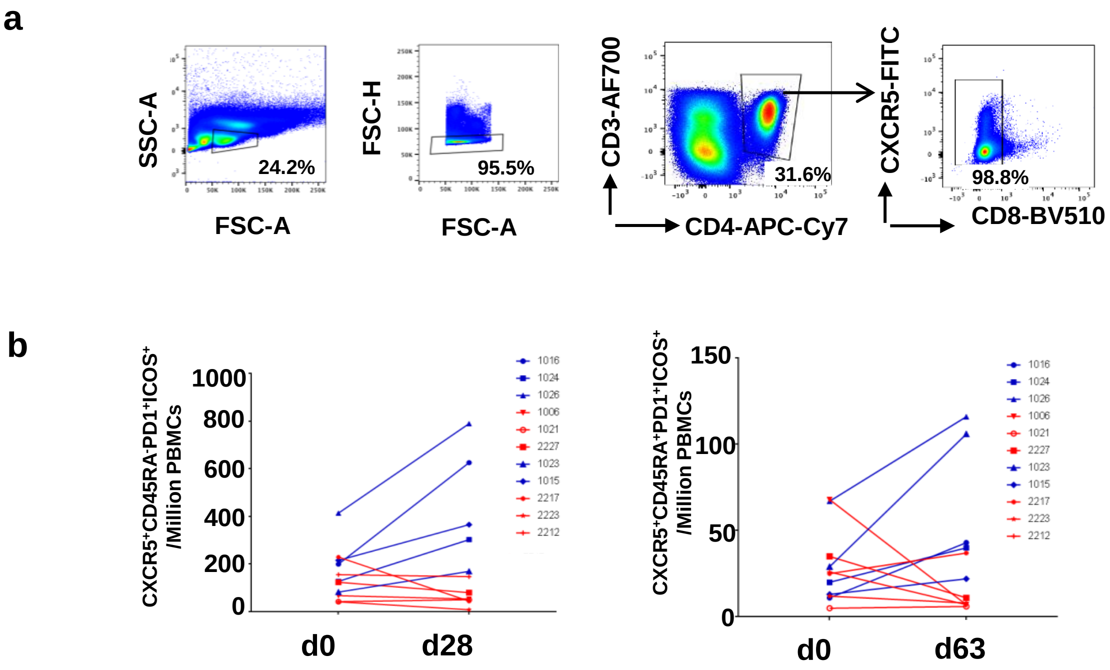

Supplementary Figure 4: Assessment of human Tfh cell responses. ***(a)*** *Gating strategy used to identify Tfh cells among human PBMCs.* ***(b)*** *Number of ICOS*$^{+}$*PD1*$^{+}$*CD4*$^{+}$*CD3*$^{+}$*CXCR5*$^{+}$*CD45RA*$^{-}$ *cells assessed among PBMCs of individual vacinees recruited to trial groups 2B (red lines) and 2C (blue lines) on d0 and d28 post ChAd63/MVA Pfs25-IMX313 vaccination. Number of ICOS*$^{+}$*PD1*$^{+}$*CD4*$^{+}$*CD3*$^{+}$*CXCR5*$^{+}$*CD45RA*$^{+}$ *cells assessed among PBMCs of individual vacinees recruited to trial groups 2B (red lines) and 2C (blue lines) on d0 and d63 post ChAd63/MVA Pfs25-IMX313 vaccination.*

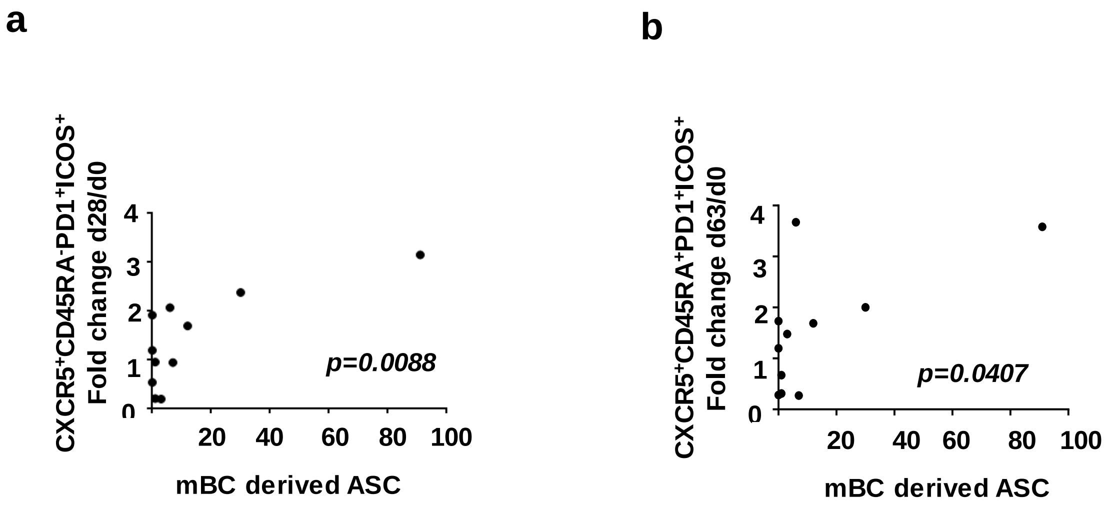

Supplementary Figure 5: Correlation between human Tfh and memory B cell responses. *Correlations are shown between numbers of human memory B cell-derived Ab secreting cells detected on d72 post vaccination and corresponding changes in numbers of either circulating ICOS*$^{+}$*PD1*$^{+}$*CD4*$^{+}$*CD3*$^{+}$*CXCR5*$^{+}$*CD45RA*$^{-}$ *cells on d28 relative to d0* ***(a)***, *or corresponding changes in numbers of ICOS*$^{+}$*PD1*$^{+}$*CD4*$^{+}$*CD3*$^{+}$*CXCR5*$^{+}$*CD45RA*$^{+}$ *cells on d63 relative to d0* ***(b)***. *P values were calculated using nonparametric Spearman's test and are shown in the graphs.*

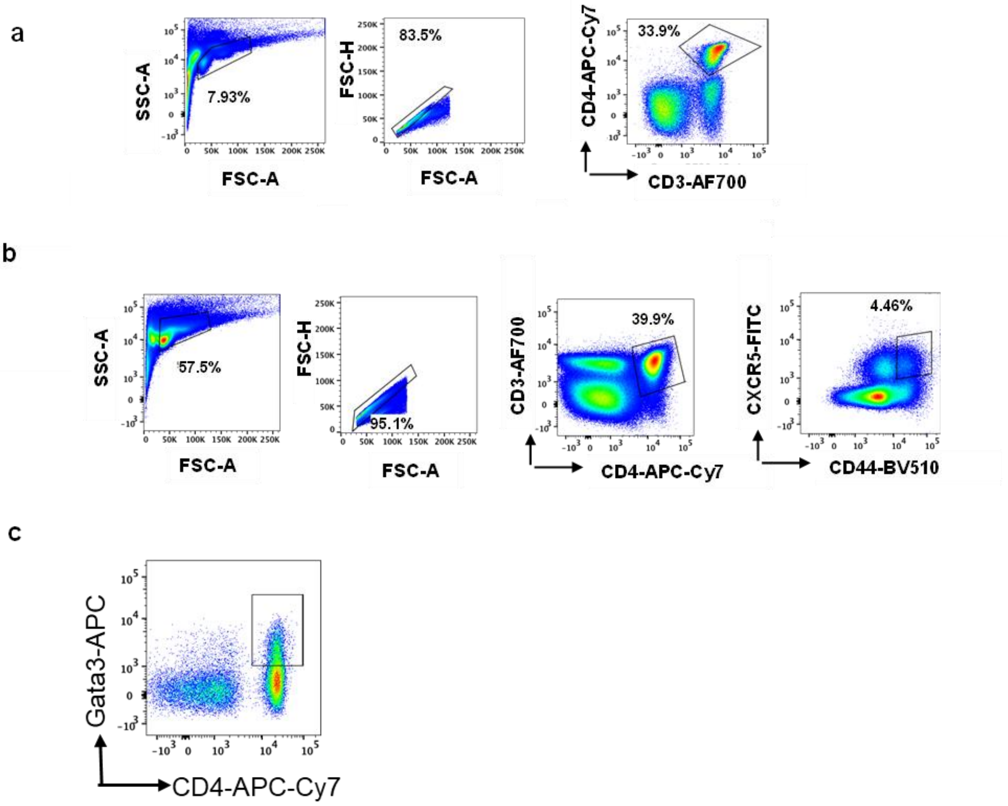

Supplementary Figure 6: Assessment of murine Tfh cell responses. *Gating strategy used to identify Tfh cells obtained from murine circulating PBMCs **(a)** or popliteal LN cell suspensions **(b).** Gata3 expression among total LN-isolated CD3⁺CD4⁺ T cells is shown in **(c)**.*

Name	Sequence		
Pfs25-1	AKVTVDTVCKRGFLIQMSGH	P1	Pfs25
Pfs25-2	RGFLIQMSGHLECKCENDLV		
Pfs25-3	LECKCENDLVLVNEETCEEK		
Pfs25-4	LVNEETCEEKVLKCDEKTVN		
Pfs25-5	VLKCDEKTVNKPCGDFSKCI		
Pfs25-6	KPCGDFSKCIKIDGNPVSYA	P2	
Pfs25-7	KIDGNPVSYACKCNLGYDMV		
Pfs25-8	CKCNLGYDMVNNVCIPNECK		
Pfs25-9	NNVCIPNECKQVTCGNGKCI		
Pfs25-10	QVTCGNGKCILDTSNPVKTG		
Pfs25-11	LDTSNPVKTGVCSCNIGKVP	P3	
Pfs25-12	VCSCNIGKVPNVQDQNKCSK		
Pfs25-13	NVQDQNKCSKDGETKCSLKC		
Pfs25-14	DGETKCSLKCLKEQETCKAV		
Pfs25-15	LKEQETCKAVDGIYKCDCKD		
Pfs25-16	DGIYKCDCKDGFIIDQESSI		
Pfs25-17	GFIIDQESSICT		
IMX313-1	GSKKQGDADVCGEVAYIQSV	P4	IMX-313
IMX313-2	CGEVAYIQSVVSDCHVPTAE		
IMX313-3	VSDCHVPTAELRTLLEIRKL		
IMX313-4	LRTLLEIRKLFLEIQKLKVE		
IMX313-5	FLEIQKLKVELQGLSKE		

Supplementary Figure 7: Pfs25-IMX313 peptide pools used for assessment of proliferative capacity of murine and human CD4$^+$ T cells.

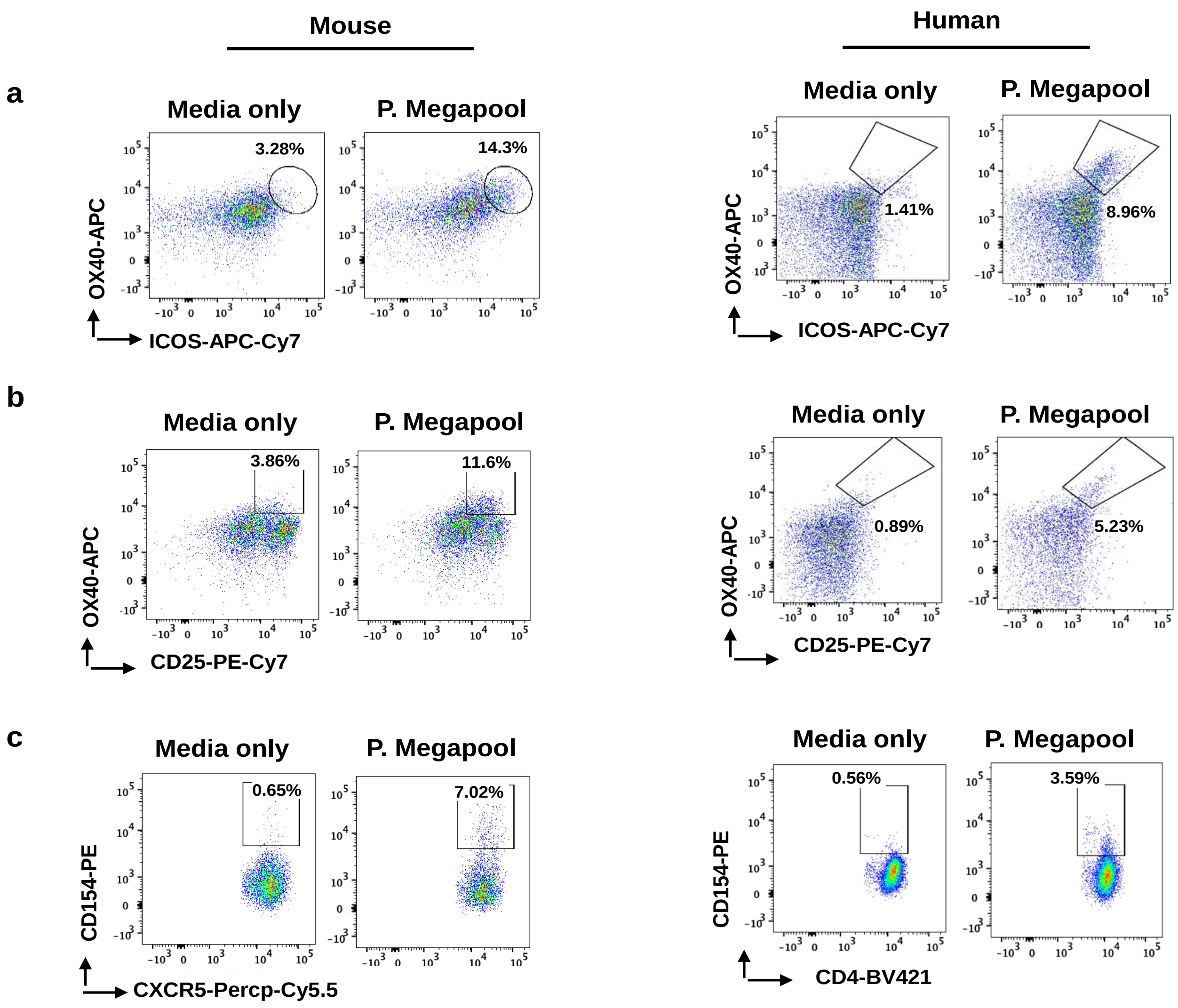

Supplementary Figure 8: Assessment of antigen specificity of murine and human Tfh cells. *Tfh cells were quantified among human PBMCs or murine splenocytes isolated on d14 after vaccination with ChAd63/MVA Pfs25-IMX313. Antigen-specific Tfh cells were identified either by OX40 upregulation in combination with ICOS*$^{+}$ ***(a)*** *or CD25 co-expression* ***(b)*** *or CD154 expression* ***(c)*** *following 18 hours of stimulation with media only or Pfs25-IMX313 peptide pools. Representative FACS plots are shown.*

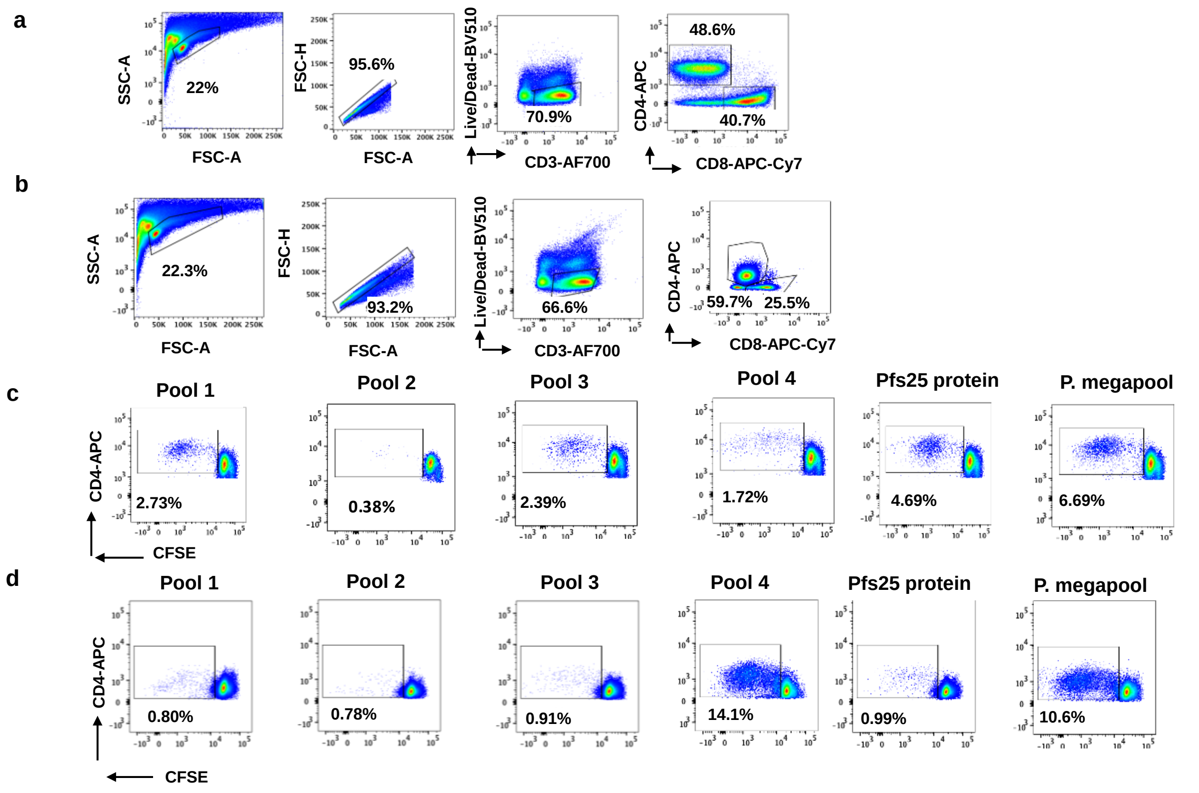

Supplementary Figure 9: Differences in proliferative capacity of murine and human CD4$^+$ T cells. (a) *Gating strategy for CD4$^+$ T cell proliferation assay by CFSE dilution among human **(a)** and murine **(b)** cells. Representative flow cytometric plots showing percentages of human **(c)** or murine **(b)** CD4$^+$ T cells that diluted CFSE in a response to various peptide pools or Pfs25 protein stimulations.*

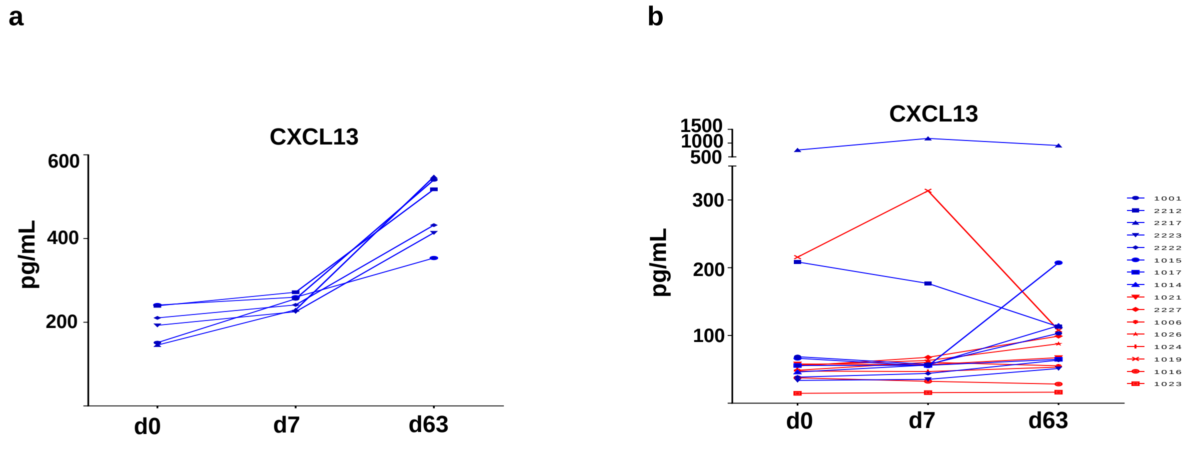

Supplementary Figure 10: Assessment of murine and human CXCL13 responses. *CXCL13 detected in individual murine **(a)** or human **(b)** serum following ChAd63/MVA Pfs25-IMX313 vaccination at indicated time points, as measured by ELISA.*

Sample name	IgG conc [ug/ml]	Average	% inhibition			
			estimate	95% CI Lo	95% CI Hi	p-value
Human serum pool	0	83.6				
4B7	94	2.9	96.6	92.2	98.6	0.001
Normal mouse Ab	750.0	22.1				
mouse sample	100.0	0.2	99.3	98.1	99.8	0.001
mouse sample	10.0	1.0	95.5	86.8	99.3	0.001
human sample	100.0	18.2	17.7	-94.1	63.5	0.641

Supplementary Table 1: Transmission-reducing efficacy of human and murine anti-Pfs25 IgG induced by the ChAd63/MVA Pfs25-IMX-313. *Table shows arithmetic mean of oocysts from 20 mosquitoes, percent inhibition of mean oocyst intensity (95% CI) and two-tailed p values testing whether %TRA is significantly different from zero.*
